# Artifacts affecting dual-energy X-ray absorptiometry and bone mineral density measurements: a case report and review of the literature

**DOI:** 10.1186/s13256-025-05353-5

**Published:** 2025-06-21

**Authors:** Kevin White, Mohamed K. M. Shakir, Christopher Nguyen, Thanh D. Hoang

**Affiliations:** 1 Division of Endocrinology, Department of Medicine, Walter Reed National Military Medical Center, 8901 Wisconsin Ave, Bethesda, MD 20889 USA; 2 Division of Endocrinology, Uniformed Services University of the Health Sciences, Bethesda, MD USA

**Keywords:** DXA artifacts, Bone mineral density, BMD

## Abstract

**Introduction:**

It is important to recognize artifacts of dual-energy X-ray absorptiometry because they may alter bone mineral density measurements. To prevent erroneous decisions and misdiagnosis regarding treatment and follow-up, bone mineral density measurement adjustments are needed.

**Case presentation:**

We present three cases in which artifacts altered the measurements of bone mineral density in the lumbar field of a dual-energy X-ray absorptiometry scan. The first case was oral contrast in the transverse colon in a 55-year-old white Hispanic American woman; the second case was kyphoplasty in lumbar spine in a 73-year-old white Hispanic American woman; and the third case was spinal fusion with vertebroplasty in a 70-year-old white European American man.

**Conclusion:**

Clinicians who interpret dual-energy X-ray absorptiometry imaging should routinely reexamine the scans and be attentive toward potential artifact involvement and other alternative origins of unreliable data.

## Introduction

Osteoporosis and osteopenia are highly prevalent in American adults over the age of 50 years, up to 55% [1, 2]. Osteopenia has a prevalence of four times greater than osteoporosis for most fractures in this population [2–4]. The hips, vertebrae, and wrists are seen as sites with the greatest incidence of fractures. Dual energy X-ray absorptiometry (DXA) is a key instrument in visualizing deficient in bone mineral density (BMD) [4]. When utilizing DXA, providers should be attentive of artifacts altering final BMD measurements. Fracture risk factors cannot solely be identified by DXA scans. In addition, a risk calculator called the Fracture Risk Assessment Tool (FRAX) has been concurrently utilized to help improve the fracture risk assessment [5]. Recommendations for treatment are clinically based on BMD T-scores. Consideration to technical accuracy and adjustments to the appearance of artifacts in DXA scans and BMD measurements are crucial in supplying reliable diagnoses and treatment recommendations. We exemplify below three cases of lumbar field DXA scans in which the appearance of artifacts derived false diagnosis.

## Case presentation

**Fig. 1:**
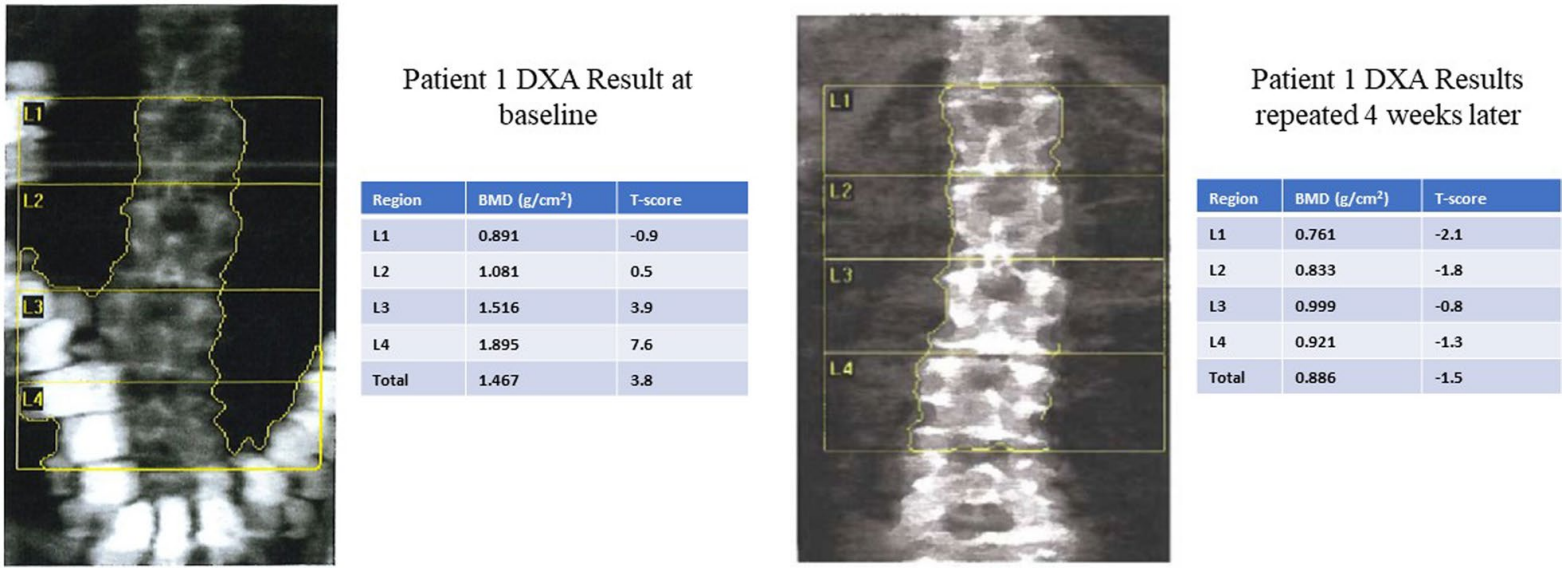
**A** DXA results of patient 1 who received oral and intravenous iodine-based contrast prior to DXA performance. **B** DXA repeated 4 weeks later

*Patient 1*: Initial DXA imaging (Fig. 1A) of a 55-year-old white Hispanic American woman with a history of rheumatoid arthritis and rheumatoid vasculitis, who recently began a treatment of high-dose prednisone therapy, revealed a lumbar spine (LS) BMD of 1.467 g/cm^2^ with a T-score of 3.8. Respective T-scores were L1 = −0.9, L2 = 0.5, L3 = 3.9, and L4 = 7.6. Bordering soft tissue, an artifact superimposed itself on the L3 and L4 fields. Prior to the first scan, the patient underwent an abdominal computed tomography with an intravenous and oral iodine-based contrast for vasculitis workup and management. Although there were marked discrepancies in BMD measurements between L1–L2 and L3–L4 fields, the diagnosis was unremarkable. However, repeated DXA scans 4 weeks later revealed resolution of the iodine-contrast artifact and the LS BMD to be 0.886 g/cm^2^ with a T-score of −1.5 (instead of the initial 3.8), consistent with a diagnosis of osteopenia (Fig. 1B).

**Fig. 2:**
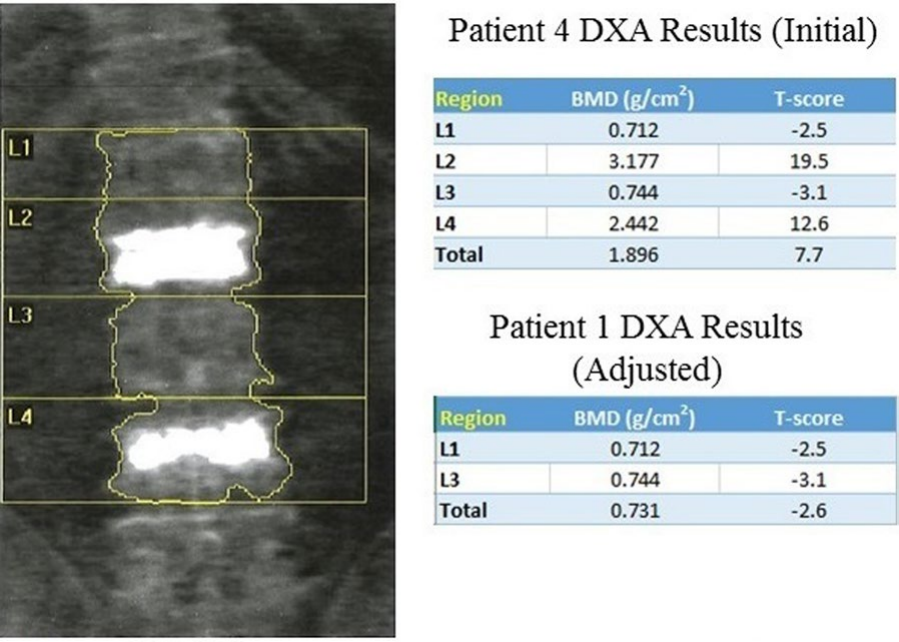
DXA results of patient 2 with kyphoplasty performed before and after adjustment

*Patient 2*: A 73-year-old white Hispanic American woman on bisphosphonates for a history of osteoporosis and a pathological lumbar compression fracture had her BMD evaluated. Initial DXA imaging (Fig. 2) exhibited LS BMD at 1.896 g/cm^2^ with a 7.7 T-score. Individual LS T-scores were L1 = −2.5, L2 = 19.5, L3 = −3.1, and L4 = 12.6. These exhibited a noticeable difference, greater than one, between L1 and L4 fields. An artifact was observed within the L2 and L4 fields. The artifacts corresponded to a kyphoplasty in L2 and L4 that the patient underwent weeks prior to presentation. When L2 and L4 were excluded, BMD was 0.731 with a T-score of −2.6. Final diagnosis was changed from normal to osteoporosis.

**Fig. 3:**
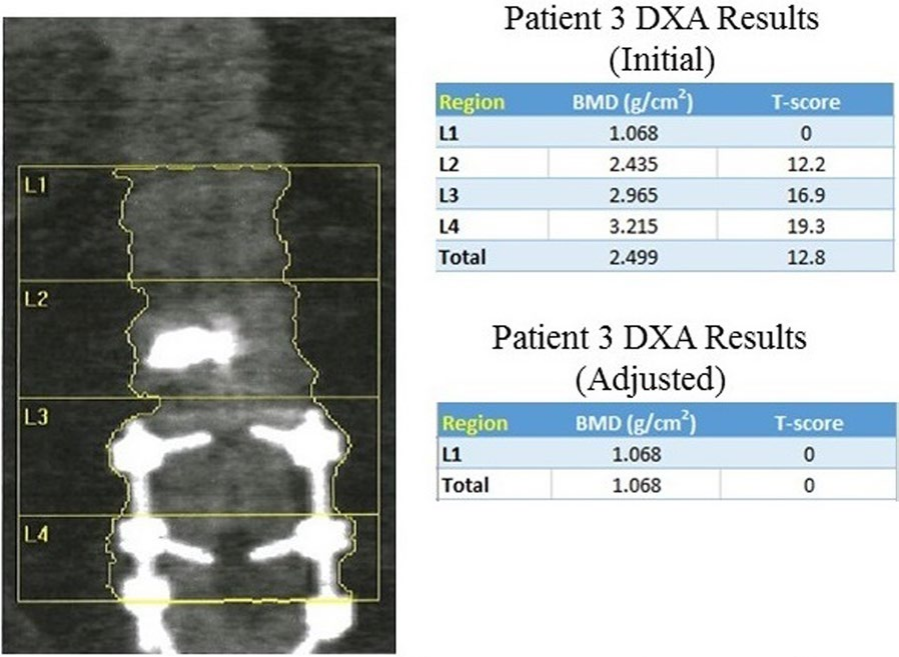
DXA results of patient 3 with a L3–L4 spinal fusion and kyphoplasty before and after adjustment

*Patient 3*: A 70-year-old white European American man with a history of multiple myeloma and chronic lower back pain, status-post spinal fusion, was seen in our clinic. DXA imaging (Fig. 3) exhibited LS BMD at 2.499 g/cm^2^ with a 12.8 T-score. Respectively, LS T-scores were: L1 = 0.0, L2 = 12.2, L3 = 16.9, and L4 = 19.3. The patient had an L2 vertebroplasty 2 years prior with a L3–5 spinal fusion that created artifacts with resulting alteration in the DXA measurements. Upon elimination of the artifacts in L2, L3, and L4 vertebrae, the total BMD was reevaluated at 1.068 g/cm^2^ with a T-score of 0.0.

## Discussion

DXA diagnoses can be altered by artifacts, with effects being more greatly observed in patient populations with low BMD [6]. Pacemakers, catheters, ports, contrast agents, bullets, aortic calcifications, and renal stones are some of the many observed artifacts known to increase the BMD measurement [6, 7]. Paraspinal soft tissue artifacts can also influence BMD evaluations as DXA measurements utilize the discrepancy in energy diminution as it passes through neighboring soft tissue and bone [6]. Tantalum surgical clips produce artifacts, also known as “black hole artifacts.” Their black appearance on imaging results in a reduction in BMD measurement. Alternatively, white densities can appear due to other surgical clips (stainless steel and titanium) and alter measurements [8]. Through a conducted experiment, it was found that BMD measurements of neighboring lumbar vertebrae can be decreased by increasing the amount of calcium tablets in paraspinal soft tissue [9].

In the first case, oral contrast in the transverse colon developed into an artifact upon further imaging. Radiodensity is pronounced by iodine-based contrast because it hinders X-ray ability to penetrate through tissue (Table 1: oral/intravenous iodine contrast section). Prior knowledge dictates that DXA scans captured shortly after dispensing intravenous and oral-iodine-based contrast increases measurements of BMD. However, imaging reproduced after 7 days generated results that do not deviate from the standard [10]. In the first case, due to contrast, BMD measurements at L3 and L4 were increased and respectively decreased at L1 and L2. Contrast being present in bordering soft tissue causes a reduction of distinction between the soft tissue and vertebrae, lowering BMD measurements.

**Table 1:** Effects of artifacts affecting bone mineral density (BMD) measurements

Causes of falsely elevated BMD	Comments
Oral and intravenous iodine contrast	Intravenous and oral contrast administration increases BMD immediately; however, DXA repeated 7 days later produces results that are not significantly changed from baseline [13]
High BMI (obesity)	The extent of the inaccuracies depends on the mean extraosseous fat to lean areal density ratio and its degree of nonuniformity within the region of interest (ROI). It is found that in many cases, inaccuracies can be as large as 20–50%, particularly in patients with osteopenia and osteoporosis, as well as in elderly patients [20]
Kyphoplasty/vertebroplasty	Kyphoplasty/vertebroplasty with polymethylmethacrylate (PMMA) increase the BMD and potentially also may improve strength in its augmented vertebrae. However, it may decrease BMD and potentially bone strength in adjacent nonaugmented vertebra above multilevel kyphoplasty
Spinal fusion	Decreases in BMD may occur above fused lumbar vertebra, related to immobilization or altered mechanics associated with arthrodesis
Spinal cord stimulator	The electrode and lead from the spinal cord stimulators lead to dense artifacts overlying the spinal lumbar area of inclusion in DXA measurement evaluation
Bone metastatic malignancies (variable effects)	Several forms of primary cancers have the potential to metastasize to local bone resulting in some forms of osteoblastic lesions, bone forming, or osteoclastic lesions, bone degenerative lesions. Commonly observed in prostate cancer and breast cancer
Degenerative joint diseases/vertebral collapse	Frequently seen in older patients, these changes manifest in the lumbar spine as end-plate osteophytosis, sclerosis, disc space narrowing, and facet joint arthropathy. Mild osteophytosis can increase lumbar spine BMD by 24% [19]
Dysplasias and dysostoses (craniodiaphysial dysplasia, osteopetrosis, melorheostosis, Engelmann disease, etc.)	In these disorders, generalized symmetric increase in bone mass is the major finding, resulting in increased BMD values
Metabolic bone disorders (hypervitaminosis [A/D], fluorosis, heavy metal poisoning, hepatitis C)	Sclerotic bone disease characterized by increased BMD may occur in hypervitaminosis A and D, lead poisoning, hepatitis C, and other conditions. In skeletal fluorosis, the ingested fluoride associates with hydroxyapatite in the bone and may cause increased lumbar BMD values

Causes of falsely decreased BMD	
Metallic implants/prothesis (tantalum surgical clips)	Also termed “black hole artifacts.” Although these surgical clips have potential to decrease the BMD at a localized area, they do not significantly decrease the L1–L4 spine BMD in a high-density spine specimen. In a low-density spine specimen, tantalum clips do have the potential to alter the BMD of a single vertebral body and the L1–L4 spinal region [10]
Laminectomy	Laminectomy is a surgical procedure that creates space by removing lamina, back part of the vertebrae that covers spinal canal, resulting in artificial appearance of natural bone loss on DXA measurements

In the second case, the patient had a pathologic fracture located in L2 and L4 and the patient underwent kyphoplasty (Table 1: kyphoplasty/vertebroplasty section). To accommodate, analysis excluded L2 and L4, but retained L1 and L3. International Society for Clinical Densitometry (ISCD) standard endorses keeping intact vertebrae and omitting vertebrae of concern, such as the presence of artifacts or regional integrity changes. Alternatively, if only one vertebra is patent, a second site is required for reaching a diagnosis [11]. The diagnosis changed from osteopenia to osteoporosis when accommodation excluded L2 and L4 from analysis of BMD measurements. 

In the third case, the surgical equipment caused an artifact in the L3 and L4 field, with a coinciding kyphoplasty causing an artifact within L2 (Table 1: spinal fusion and kyphoplasty/vertebroplasty sections). Upon DXA imaging, distinction between L3 and L4 due to the presence of an artifact could not be made. Once unimpeded, the total BMD returned to baseline. However, due to the presence of multiple artifacts, only the vertebra was evaluated as sufficient for inclusion. With artifact occurrence, guidelines for alternative site evaluation were conducted.

FRAX scores may be sufficient without BMD determination, yet also have a comparable assessment to BMD solely [5, 12]. Utilizing FRAX is advantageous in patients with a permanent lumbar artifact. This is like our third case because BMD from the femoral neck is included. Special cohorts may present difficulties in assessing fracture risk. For instance, women past their child-bearing years have the highest rate of osteoporotic fractures. It should be noted that usage of DXA scans in this cohort have been highly debated [13, 14]. Fracture risks in patients receiving glucocorticoids are dose-dependent, and FRAX may underestimate risk since it incorporates the equivalent of a prednisone dose of 2.5–7.5 mg/day instead of the higher doses utilized clinically [15]. There are challenges in evaluating risk for rheumatoid arthritis with intermittent use of high-dose glucocorticoids [16]. As seen in rheumatoid arthritis patients, lumbar compression fractures are particularly commonplace in patients with inflammation-mediated osteoporosis and can hide the underlying diagnosis of osteoporosis. [17]. In the same vein, bone integrity decline at the distal radius may also be responsible for over detection of osteoporosis [17].

Additionally, DXA user and/or machine should be acknowledged as potential alternative sources of error to acknowledge outside of artifacts [7]. Other possible errors can come from comparing scans from separate medical practices. Lastly, variation in the region of interest (ROI) on serial images can also induce errors such as examining the lumbar ROI more caudally by one vertebral body and using L2–L5 for analysis [7].

## Conclusion

Clinicians who interpret DXA imaging should routinely reexamine the scans and be attentive toward potential artifact involvement and other alternative origins of unreliable data (Table 1) [8–10, 17–20]. FRAX utilization is exceptionally advantageous in patients with permanent lumbar artifacts.

## Data Availability

All data have been included in the tables and figures; there are no additional data that were presented or discussed but not included in manuscript.

## Abbreviations

BMD: Bone mineral density
DXA: Dual-energy X-ray absorptiometry
FRAX: Fracture risk assessment tool
LS: Lumbar spine
LAGB: Laparoscopic adjustable gastric banding
